# New Insights on Developmental Dyslexia Subtypes: Heterogeneity of Mixed Reading Profiles

**DOI:** 10.1371/journal.pone.0099337

**Published:** 2014-06-11

**Authors:** Rachel Zoubrinetzky, Frédérique Bielle, Sylviane Valdois

**Affiliations:** 1 Centre Référent de Diagnostic des Troubles du Langage et des Apprentissages, Pôle Couple-Enfant, Centre Hospitalier Universitaire, Grenoble, France; 2 Laboratoire de Psychologie et NeuroCognition, CNRS, UMR 5105, Université Grenoble Alpes, Grenoble, France; Utrecht University, Netherlands

## Abstract

We examined whether classifications based on reading performance are relevant to identify cognitively homogeneous subgroups of dyslexic children. Each of the 71 dyslexic participants was selected to have a mixed reading profile, i.e. poor irregular word and pseudo-word reading performance (accuracy and speed). Despite their homogeneous reading profile, the participants were found to split into four distinct cognitive subgroups, characterized by a single phonological disorder, a single visual attention span disorder, a double deficit or none of these disorders. The two subgroups characterized by single and contrasted cognitive disorders were found to exhibit a very similar reading pattern but more contrasted spelling performance (quantitative analysis). A qualitative analysis of the error types produced in reading and spelling provided some cues about the participants' underlying cognitive deficit. The overall findings disqualify subtyping based on reading profiles as a classification method to identify cognitively homogeneous subgroups of dyslexic children. They rather show an opaque relationship between the cognitive underpinnings of developmental dyslexia and their behavioral manifestations in reading and spelling. Future neuroimaging and genetic studies should take this issue into account since synthesizing over cognitively heterogeneous children would entail potential pitfalls.

## Introduction

Developmental dyslexia is a specific learning disability characterized by an unexpected difficulty in learning to read in children who have at least average intelligence, who do not have general learning difficulties, and whose reading problems are not due to extraneous factors that might interfere with learning to read, such as sensory acuity deficits, severe emotional problems, acquired brain damage or inadequate educational opportunity [1]. The dyslexic population is highly heterogeneous so that different dyslexic children may exhibit different reading profiles and their poor reading performance can be associated with different cognitive impairments and different brain dysfunctions [2]–[5]. Classification systems have been proposed to reduce this heterogeneity and identify more homogeneous subgroups [6], [7]. A very popular subtyping approach is based on the recognition of different reading profiles as a way to distinguish cognitively distinct subtypes of developmental dyslexia.

Individual differences in developmental dyslexia have been conceptualized using dual route models [8], [9]. The case study approach revealed the existence of two distinct reading profiles of developmental phonological and surface dyslexia. Prototypical cases of developmental phonological dyslexia show selective difficulties in pseudo-word reading but relatively preserved irregular word reading [10]–[15]. This reading profile is viewed as resulting from the inability of dyslexic children to acquire grapheme-phoneme conversion rules due to a phonological disorder. The converse reading pattern characterizes developmental surface dyslexia in which pseudo-word reading is preserved but irregular word reading is selectively impaired [15]–[21]. Within the dual route framework, this profile is viewed as following from the selective impairment of the lexical pathway due to a specific orthographic processing disorder.

Through group studies, subtypes of reading disability have been identified based on the relative imbalances on the tasks of irregular word and pseudo-word reading. Regression techniques were then used to identify dyslexics with larger than expected discrepancies between irregular word and pseudo-word reading, based on the linear relationship between irregular words and pseudo-words in the control group. Using this procedure, two groups of phonological and surface dyslexic children have been identified as compared to age-matched controls. However, only about a third of the children exhibited strict discrepancies between irregular word and pseudo-word reading (i.e., poor performance on one type of items but not the other); most were impaired on both types of items, thus showing a pattern of mixed dyslexia. The preponderance of mixed reading profiles in the dyslexic population was estimated from 53% to 76% depending on the studies [8], [22]–[26] (see [27] for a cross-language review).

Although classifications based on reading patterns were initially proposed to identify cognitively homogeneous subgroups of developmental dyslexia, there is no strong evidence for such a direct relationship. To the contrary, comparative studies of the phonological-like and surface-like groups as defined through regression analyses failed to show contrasted cognitive profiles. In fact, a phonological disorder was reported not only in the phonological dyslexia subtype but also in the group of children identified through regression methods as having a surface-like profile [28], [24], [26]. Moreover, most studies focused on the phonological and surface dyslexia profiles; none explored what cognitive disorders were associated with the most frequent mixed dyslexia subtype and none provided a straightforward account of why this pattern predominates in the dyslexic population. The main purpose of the present study was to investigate the cognitive underpinnings of the dyslexic group characterized by a mixed reading pattern. We focused on the mixed reading pattern because of its prevalence in the dyslexic population and because of the difficulty of theoretical models to account for its overrepresentation. We will show that mixed dyslexia is associated with a variety of distinct cognitive disorders, against the hypothesis of a one-to-one mapping between reading patterns and cognitive disorders. To explore more in-depth the relationship between cognitive disorders and their behavioral manifestations, we will then focus on the two subsets of dyslexic children with a single but contrasted cognitive disorder to explore whether contrasted cognitive impairments differently modulated reading and spelling performance. More generally, the present study will address the issue of the relationship between cognitive disorders and their behavioral manifestations. We will provide new insights on the relevance of dyslexia subtypes while questioning the validity of classifications based on reading patterns.

### 1. Classical accounts of mixed reading patterns

Typically, dual route models interpret mixed dyslexia as cumulating the disorders of phonological and surface dyslexia, thus showing dysfunctions of both the analytic and lexical reading pathways [22]. However, dual route models do not provide any straightforward account of the higher probability for a double deficit rather than a single in the dyslexic population. The preponderance of double deficits seems rather implausible statistically and should be cognitively and neurologically explained if observed. The high incidence of mixed reading patterns is thus challenging for dual route models.

Subsequent theoretical advances led to the new hypothesis that mixed dyslexia might follow from a single phonological disorder. Indeed, Share's self-teaching hypothesis postulates that the development of orthographic knowledge mainly relies on phonological processing abilities [29]–[31]. The child who encounters a word he never read before will decode it by applying grapheme-phoneme conversion rules. Each successful decoding will provide the opportunity to gradually strengthen word orthographic knowledge in long-term memory, thus leading to acquire the large sight vocabulary required for rapid recognition of words and fluent reading. The self-teaching hypothesis suggests that dyslexic children who show a phonological disorder should exhibit a pseudo-word reading disorder as a direct consequence of their poor decoding skills but they should further show a (regular and irregular) word reading problem due to the impact of their poor decoding on the self-teaching device. Theoretical evidence for a central role of phonological processing in the building up of the two (analytic and lexical) reading pathways is well in line with the hypothesis that developmental dyslexia follows from a core phonological disorder [32], [33]. This hypothesis is further supported by behavioral and modeling data. At the behavioral level, phonological and decoding skills appear as key agents of learning to read [34] and strong predictors of successful orthographic learning [31]. Within the framework of PDP connectionist models, Harm & Seidenberg [35] showed through simulations that a mild phonological disorder primarily impacted pseudo-word reading while additional word reading problems occurred following a more severe phonological disorder (also [22], [23]). Thus, both the self-teaching hypothesis and modeling data from PDP connectionist networks support the hypothesis that mixed dyslexia could follow from a single phonological disorder. However, PDP connectionist models as dual route models cannot straightforwardly account for the prevalence of mixed reading profiles, unless explaining why phonological problems should be more often severe rather than moderate or mild in the dyslexic population.

### 2. The multitrace memory (MTM) model account of mixed reading profiles

The MTM model [36] postulates that developmental dyslexia can result from two distinct disorders, either a phonological or a visual attention (VA) span disorder, or both. The phonological disorder would primarily impact pseudo-word reading and further prevent normal acquisition of orthographic knowledge, as postulated by the self-teaching account, so that a single phonological disorder can result in a mixed dyslexia profile. However, the most innovative prediction of the model is that a VA span reduction should also result in developmental dyslexia, and sometimes in a mixed reading profile.

Within this framework, the VA span corresponds to the amount of orthographic information that can be simultaneously processed when reading [2]. In delineating the number of letters that are simultaneously processed at each step of the reading process, the VA span is involved in the processing of all relevant multi-letter orthographic units from graphemes to whole words [37]. Accurate reading of irregular words and rapid recognition of regular words require a large VA span that encompasses all the letters of the word sequence. A VA span reduction that prevents the entire word letter string to be simultaneously processed may thus result in poor accuracy performance on irregular words and slowed reading speed on words, thus generating a reading profile of surface dyslexia [37]. In line with this hypothesis, a VA span reduction was reported in some prototypical cases of surface dyslexia [38], [39], [15], [5]. Regardless of dyslexia subtypes, group studies further revealed that the VA span disorder contributed to the poor reading outcome of dyslexic children independently of their phoneme awareness skills [2]. A VA span disorder was reported in dyslexic children who demonstrated no phonological problems [2], [5], [39]–[42], while other dyslexic participants showed a phonological disorder but preserved VA span [2], [12]. Both the MTM model and behavioral data suggest that the VA span acts as a second core disorder in developmental dyslexia and is a key agent of reading acquisition. Indeed, a study carried out on a large sample of typically developing children from first, third and fifth grades showed that the VA span predicts variations in learning to read at all grades, independently of the influence of phoneme awareness [43]. Moreover, this study revealed that VA span abilities contributed not only to word reading but also to pseudo-word reading performance (see also [2]). Indeed, pseudo-word reading requires the VA span to be large enough to process in parallel all the letters of relevant sublexical units (as multi-letter graphemes or syllables). A reduced VA span could thus result in poor performance in both word and pseudo-word reading, thus leading to a mixed reading profile.

This prediction was first assessed through the case study of Martial, a 9 year-old child with a severe mixed dyslexia [42]. Martial was found to have remarkably preserved phonological abilities (i.e., good oral language, good phoneme awareness and good verbal short-term memory skills) but a severely reduced VA span. When asked to report as many letters as possible from a briefly presented 5-consonant letter string, he could only identify two letters out of 5 at the expected level. When asked to process briefly presented words of different length (from 3 to 9 letters long), he could only report 3 letters accurately on average whatever word length. Thus, Martial's case study exemplifies the MTM model's prediction that mixed dyslexia can follow from a single VA span disorder. Simulations carried out within the connectionist network that implemented the MTM model confirmed that a selective VA span reduction could affect both real word and pseudo-word reading [36].

In fact, the MTM model provides a multifactorial account of mixed reading patterns in developmental dyslexia. The model posits that a single phonological disorder can result in a mixed reading profile if further impacting the self-teaching device. It further emphasizes the importance of the VA span as a second key component of learning to read and predicts that a mixed reading profile can follow from a single VA span disorder but preserved phonological skills. Of course, a double deficit of phonological processing and VA span would further result in a mixed reading profile. The MTM model thus predicts that the mixed reading pattern in developmental dyslexia may follow from three distinct cognitive dysfunctions: a single phonological disorder, a single VA span disorder or a double deficit. Evidence for different cognitive disorders in mixed dyslexia is a very important issue that may provide a straightforward account of the prevalence of this reading profile in the dyslexic population.

### 3. Overview of the current study

The current study was carried out by reference to the MTM model of reading, thus discarding the potential influence of other mechanisms (as rapid automatized naming) not involved in the model. Our aim was to assess whether each of a single phonological disorder or a single VA span disorder or both disorders may result in a mixed reading profile. Overall, our working hypothesis will be that mixed dyslexia is characterized by a causal heterogeneity, i.e. a many-to-one mapping across the cognitive and behavioral levels [44]. Within this framework, each phonological and VA span disorder is viewed as necessary and sufficient to result in a mixed dyslexia profile. Note that the phonological and VA span processes are components of the reading system that play an independent role in reading acquisition [2], [43], [37]. As expected within interactive frameworks, simulations already showed that mixed reading profiles can follow from a single phonological disorder [35] or a single VA span disorder [36]. We therefore expected each of these cognitive disorders to characterize subsets of children with a mixed dyslexia profile, whatever the associated symptoms (comorbidity) they might otherwise exhibit.

For this purpose, the current study focused on a group of dyslexic children chosen to have a mixed reading profile at the individual level - i.e., poor (regular and irregular) word and pseudo-word reading performance (accuracy and speed) - and for whom phoneme awareness and VA span abilities were investigated. The first section of the paper (Part 1) will focus on the whole population to explore whether this mixed reading profile dyslexic population falls into distinct cognitive subgroups characterized by either a single phonological disorder, or a single VA span disorder, or both disorders. The methodology we used is very close to that of Bosse et al. [2]. In this previous paper however, the issue of multiple cognitive disorders was explored in a dyslexic population that was not a priori selected to have a specific reading profile. Accordingly, this study provided no insight on the relationship between cognitive disorders and reading profiles, which is specifically addressed in the current study. Contrary to Bosse et al's results [2], the classical view of mixed dyslexia (based on dual route models) would predict a large predominance of double deficits (poor phoneme awareness AND reduced VA span) in our sample of dyslexic individuals. The causal heterogeneity hypothesis does not make such a strong prediction within the framework of interactive models and reading acquisition. In this framework, a single deficit may be detrimental for the building up of the whole reading system whether it selectively affects phonological processing or the VA span. Evidence for distinct cognitive disorders in a population characterized by a homogeneous reading profile of mixed dyslexia will be taken as first evidence that subtyping based on reading profiles is not relevant to identify cognitively homogeneous subtypes of developmental dyslexia.

In a second section (Part 2), we will focus on two groups of dyslexic children selected from the whole population to have a single phonological or a single VA span disorder but similar chronological age and reading level. We will first compare their VA span and phoneme awareness performance to that of two control groups matched for chronological age and reading age. Our main purpose will be to show that the group with a pure VA span disorder remains impaired when compared to younger children of the same reading level (similar findings are further expected with respect to phoneme awareness). We will then conduct quantitative and qualitative analyses to explore to what extent these cognitively distinct subgroups differ at the behavioral level. The quantitative analysis will focus on performance in reading and spelling that were both expected to suffer from either a VA span disorder or a phonological disorder [45], [15], [42]. In particular, we will investigate whether a pure phonological disorder yields poorer performance in pseudo-word reading than a pure VA span disorder, as poor pseudo-word reading is typically viewed as reflecting poor phonological skills. In the same way, a phonological disorder was expected to prevent accurate pseudo-word spelling which should be mainly preserved following a VA span disorder. In contrast, similar reading and spelling performance in the two groups of dyslexic children with distinct cognitive disorders would be strong evidence that cognitively distinct subtypes of developmental dyslexia do not necessarily yield distinct reading/spelling profiles. We will lastly conduct a qualitative analysis to explore whether contrastive cognitive disorders result in distinct error patterns. Phonological errors are expected in the context of a phonological disorder while regularization errors in reading and phonologically plausible errors in spelling should predominate in cases of dyslexia related to a VA span deficit but preserved phonological skills. In line with previous evidence from a single case study [42], we will further expect visual errors to predominate in the group with a single VA span disorder; parsing errors due to partial decoding of the whole letter-string that forms complex graphemes (e.g., “AIN” read “A”-“IN” instead of “AIN” /ê/) will be in particular expected following limited visual attention resources.

Overall, there is substantial evidence that the dyslexic population is cognitively and neurophysiologically heterogeneous. We will here explore whether reading profile subtyping is relevant or not to select homogeneous groups of dyslexic children from which meaningful conclusions at the cognitive, neurobiological, genetic, and therapeutic level can be made.

## Part 1: Whole population investigation

### 1. Method

#### 1.1. Participants

One hundred and forty-two French native speakers took part in this experiment: 71 dyslexic children with mixed dyslexia and 71 control children. The research was approved by the local ethic committee of the Université de Grenoble. All children and their parents or guardians have provided their written informed consent to participate in this study. Dyslexic participants were recruited at the center for learning disabilities of Grenoble University Hospital where they received a complete medical, psychological and cognitive assessment. All participants had normal IQ (exclusion if Progressive Matrices <25° percentile or if VCI and PRI <85 on the Wechsler Intelligence Scale for Children IV); they attended school regularly and none of them had any history of neurological illness or brain damage. Dyslexic children were excluded from the study if their reading disorder was associated with a specific language impairment or an attention disorder with or without hyperactivity. Each selected dyslexic child showed poor performance (at least 1.6 SD below the norm) in both irregular word and pseudo-word reading (accuracy or speed) at the individual level (median SD below the norm of the selected SDs, on accuracy score or speed, for irregular word  = −4.4 SD, for pseudo-words  = −3.8 SD). As a group, dyslexic children achieved a mean reading age of 7 years and 6 months on the Alouette Reading Test [46], corresponding to a reading delay of more than three years on average. Normally developing children were monolingual French speakers recruited from schools of the Grenoble urban area. They reported no history of oral language or reading disorder. None had repeated a grade. The two groups of dyslexic participants (mean age  = 10 years 5 months, SD  = 22 months) and normally developing control children (mean age  = 10 years 5 months, SD  = 14 months) were matched on chronological age (t (141)  = −0.33, p = .74) but they differed in reading age (10 years and 5 months, SD  = 22 months for control children, 7 years and 3 months, SD  = 6 months for dyslexics children, t (141) = 13.8, p<.0001). Descriptive data of the groups is provided on Table 1.

**Table 1:** Descriptive data.

	CA controls		Dyslexics			
	Mean (SD)	Range	Mean (SD)	Range	t (140)	*p*
Age (months)	125,1 (13,7)	99–146	125,9 (16,4)	98–175	−0,33	*0,744*
Reading Age (months)	125,2 (22,4)	88–171	87,4 (5,6)	78–102	13,80	*<0,001*
RW score (/20)	19,3 (0,8)	17–20	15,6 (2,7)	6–20	10,94	*<0,001*
RW time (sec)	16,1 (4,9)	8–30	49,2 (20,6)	18–159	−13,15	*<0,001*
IW score (/20)	17 (2,5)	10–20	9,8 (3,5)	1–20	14,08	*<0,001*
IW time (sec)	18 (6,9)	7–41	60,5 (29,2)	18–229	−11,92	*<0,001*
PW score (/20)	17,8 (1,4)	14–20	11,5 (3,2)	3–18	15,12	*<0,001*
PW time (sec)	22 (6,3)	11–43	57,7 (20,4)	19–192	−14,09	*<0,001*
Deletion (%)	84,4 (16,8)	40–100	69,4 (21,2)	30–100	4,69	*<0,001*
Segmentation (%)	57,3 (27,5)	6–100	53,1 (28,7)	0–100	0,88	*0,383*
Acronyms (%)	84,5 (15)	50–100	66,3 (24,3)	0–100	5,36	*<0,001*
Letter identification (/50)	44,4 (6,3)	26–50	39,2 (7,5)	17–50	4,51	*<0,001*
Whole report (%)	83,9 (9,6)	63–100	70 (11,6)	46–90	7,81	*<0,001*
Partial report (%)	87,9 (8,1)	64–100	75 (13,1)	38–100	7,00	*<0,001*

Mean scores, standard deviation (SD) and ranges of regular word (RW), irregular word (IW), and pseudo-word (PW) reading, phonological and visual attention span tasks for the dyslexic and chronological age matched (CA) control participant.

#### 1.2. Material and procedure

The test session included three reading tasks, three phoneme awareness tasks and two visual attention span tasks, plus a control single letter identification task. The dyslexic children were tested individually at the center for Learning Disabilities of Grenoble University Hospital. The control children were tested individually in one or two sessions in a quiet room of their school. The phonological, visual attention span and reading tests were presented in a random order that varied from one child to the other.

Reading skills were assessed using tasks of isolated word and pseudo-word reading, taken from the ODEDYS neuropsychological battery [47]. Participants were administered six lists of 20 items each for a total of 40 regular and 40 irregular words of high and low frequency (HF and LF), and 40 pseudo-words. The regular and irregular word lists were matched for letter and syllable length, grammatical class and frequency. The 40 pseudo-words were legal pseudo-words without lexical neighbors. The participants were instructed to read aloud each of the six lists of 20 items as quickly and as accurately as possible. Both accuracy and reading speed were taken into account. A composite score was created from performance on the two lists of pseudo-words and the two HF and LF lists for the regular and irregular words to obtain an average reading accuracy (maximal  = 20) and an average reading speed performance.

The phoneme awareness was assessed using a phoneme deletion task and a phoneme segmentation task which were taken from [43], and an acronyms task from the BELEC battery [48]. For each task, the participants were given a set of practice items for which they received feedback. No feedback was provided on the experimental items. The dependent variable was the percentage of correct responses.

In the phoneme deletion task, the participants had to delete the first sound of a spoken word and produce the resulting pseudo-word (e.g., “outil” /uti/: /ti/; “placard” /plakaR/: /lakaR/). Twenty experimental words were presented: 7 began with a vocalic phoneme corresponding to a multiple letter grapheme so that the omission of the first letter (instead of the first phoneme) yielded incorrect responses, 9 began with a consonantal cluster, 4 with a singleton.

In the phoneme segmentation task, the participants had to successively sound out each phoneme of a spoken word (e.g. /kado/ ‘cadeau’ gift: /k/- /a/- /d/- /o/). Fifteen words were presented that were made up of 4 phonemes on average (range 3–5).

In the acronyms task, two words were successively pronounced (one word per second). The children were asked to extract the first phoneme of each word and blend them to produce a new syllable (e.g. “photo” “artistique” /foto/-/aRtistik/ says /fa/). The test comprised 10 series of two words made up of 4.4 phonemes on average (range 2–8). Seven words began with a phoneme corresponding to a digraph so that an erroneous word was generated if the first letter was extracted instead of the first phoneme (response /pa/ instead of /fa/ if orthographically biased in the above example).

Two tasks of global and partial letter report were used to assess VA span abilities. A task of single letter identification threshold was further administered to control for single letter processing. Although they involve a verbal response and use verbal stimuli, there is strong evidence that the letter report tasks primarily address visual attention span abilities. Indeed, performance on these tasks is not sensitive to verbal memory load [40] and not affected by concurrent articulation [49]. Dyslexic children with poor letter report performance are similarly impaired when using non-verbal tasks and non-verbal material [50]. Lastly, the VA span involves attentional but not phonological brain regions [51], [41], [5], [52]. Moreover, similar brain activations are observed regardless of the verbal or non-verbal nature of the stimuli to be processed [53]. The letter report tasks were displayed on a PC computer using E-prime software (E-prime Psychology Software Tools Inc., Pittsburgh, USA).

For the global and partial letter report tasks, random five letter-strings (e.g., RHSDM; angular size  = 5.4°) were built up from 10 consonants (B, P, T, F, L, M, D, S, R, H). The strings contained no repeated letters. The 5-consonant strings never matched the skeleton of a real word (e.g.: FLMBR for FLAMBER “burn”). Two subsequent letters never corresponded to a French grapheme (e.g. PH, TH) or a frequent bigram in French (e.g. TR, PL, BR). The letters were presented in upper case (Arial, 7 millimeters high) in black on a white background. The distance between adjacent letters was of 0.57° in order to minimize crowding. Twenty 5-letter strings were displayed in Global Report. Each letter was used ten times and appeared twice in each position. Fifty random 5-letter strings were used in Partial Report. Each letter occurred 25 times (5 times in each position). At the beginning of each trial, a central fixation point was presented for 1000 ms followed by a blank screen for 50 ms. Then, a letter-string was displayed at the center of the screen for 200 ms, a duration which corresponds to the mean duration of fixations in reading, long enough for an extended glimpse, yet too short for a useful eye movement. In the Global report condition, children had to report verbally as many letters as possible immediately after the string disappeared. In Partial Report, a vertical bar indicating the letter to be reported was displayed 1.1° below the target letter, at the offset of the letter-string. Each letter was used as target once in each position. Participants were asked to report the cued letter only. In both tasks, the experimenter pressed a button to start the next trial after the participant's oral response. The experimental trials were preceded of 10 training trials for which participants received feedback. No feedback was given during the experimental trials. The dependent measure was the percentage of letters accurately reported (identity not location) across the 20 trials in Global report or across the 50 trials in Partial report.

In the letter identification control task, each of the 10 letters used in the report tasks were randomly presented (5 times each) with the same physical characteristics as in the experimental tasks, at 5 different presentation durations (33, 50, 67, 84 and 101 ms). At the offset of the letter, a mask (13 mm high, 37 mm wide) was displayed for 150 ms. Participants were asked to name each letter immediately after its presentation. The test trials were preceded of 10 practice trials (2 for each presentation time) for which participants received feedback. Children were excluded when the maximal score of 10 good responses was not reached at the maximal presentation duration of 101 ms. The total score was the sum of scores at each display duration.

#### 1.3. Design and analyses

A correlation analysis was first conducted on the measures of Age, Reading, Phonological Awareness and Visual Attention Span, for the whole population of dyslexic and control children (Bonferroni correction: *p*<0.05/(14*14)). When required, data were log-transformed to meet normality assumption. Second, to reduce the data set before exploring the concurrent predictors of reading skills among mixed dyslexic and normally developing children, we conducted a principal components analysis with varimax rotation on the data from the 3 phonological tasks and the 2 visual scores. All factor loadings greater than /0.70/ were used for interpretation. A hierarchical regression analysis from the factor scores derived on the basis of the principal components analysis was used to explore the contribution of each factor to reading skills. We then studied the participant's repartition according to their factorial coefficients and we distinguished cognitively based subgroups among the mixed dyslexia population.

### 2. Results

#### 2.1. Overview of the participants' performance

Performance of the dyslexic and control participants on each task of the assessment battery is provided in Table 1. The dyslexic group shows poorer performance than the control group on all the reading and VA span tasks. The dyslexic participants further show lower performance on two of the phoneme awareness tasks, namely deletion and acronyms but not in phoneme segmentation. On average, they also identified fewer briefly presented single letters than normal readers.

#### 2.2. Correlation analyses

Results of correlation analyses carried out on the measures of reading, phonological skills and VA span - with and without controlling for age (CA) and letter identification skills (Letter Id.) - are provided in Table S1. Strong correlations were found between the measures thought to reflect the same cognitive processes. However as typically found, performance on the phonological and VA span tasks was mainly unrelated, suggesting that these tasks tap different cognitive processes. In line with our previous findings, both phonological and reading skills on one hand and VA span and reading skills on the other hand correlated significantly.

#### 2.3. Principal components analyses

To reduce the data set before exploring the concurrent predictors of reading skills among mixed dyslexic and control children, we computed a principal components analysis with varimax rotation on the two VA span and the three phonological tasks. The analysis revealed a two-factor solution. The first factor accounted for 30.8% of the variance and received high loadings from the deletion, segmentation and acronyms tasks (from 0.76 to 0.80 - called phonological factor hereafter). The second factor with high loadings from the global and partial report tasks (0.91 and 0.92 - called VA span factor here after) accounted for a further 31.4% of the variance.

The individual phonological and VA span factorial coefficients were then used as potential predictors of reading subskills. Two different sets of hierarchical regressions were carried out. In all cases, chronological age and letter identification were entered as control tasks at step 1. We then forced the entry of either the phonological factor or the VA span factor at step 2 to assess the unique contribution of each factor to the different reading measures at step 3. Results of these analyses are shown in Table 2.

**Table 2:** Results of hierarchical regressions.

	R^2^ Change					
Factor	RW score	RW time	IW score	IW time	PW score	PW time
1. Control	.073**	.108***	.142***	.127***	.064**	.103***
2. Phonological	.204***	.065**	.220***	.066***	.156***	.031*
3. VA span	.186***	.302***	.262***	.261***	.277***	.292***
						
2. VA span	.188***	.303***	.264***	.262***	.279***	.293***
3. Phonological	.203***	.063***	.218***	.065***	.155***	.030**
						
Total	.465***	.475***	.624***	.454***	.497***	.427***

Contribution of each of the phonological and VA span factors to regular word (RW), irregular word (IW) and pseudo-word (PW) reading, accuracy and speed. The First Step corresponds to the forced entry of the two control variables (CA and letter identification).

*** p<.001 ** p<.01 *p<.05.

The whole model accounted for more than 40% of the variance in each reading task (from 42.7% to 62.4%). The phonological factor and the VA span factor both independently contributed to accuracy (from 15.5% to 27.7%), and speed performance (from 3% to 30.2%) on all reading sub-skills. In line with the self-teaching account, phonological skills independently contributed to irregular word reading (22% accuracy, 6% speed). Interestingly, the VA span factor was found to account for 28% of unique variance in pseudo-word reading accuracy (29% for speed), suggesting a non-trivial contribution of this “visual” factor to pseudo-word reading.

#### 2.4. Identification of cognitively based dyslexic subtypes

**Figure 1:**
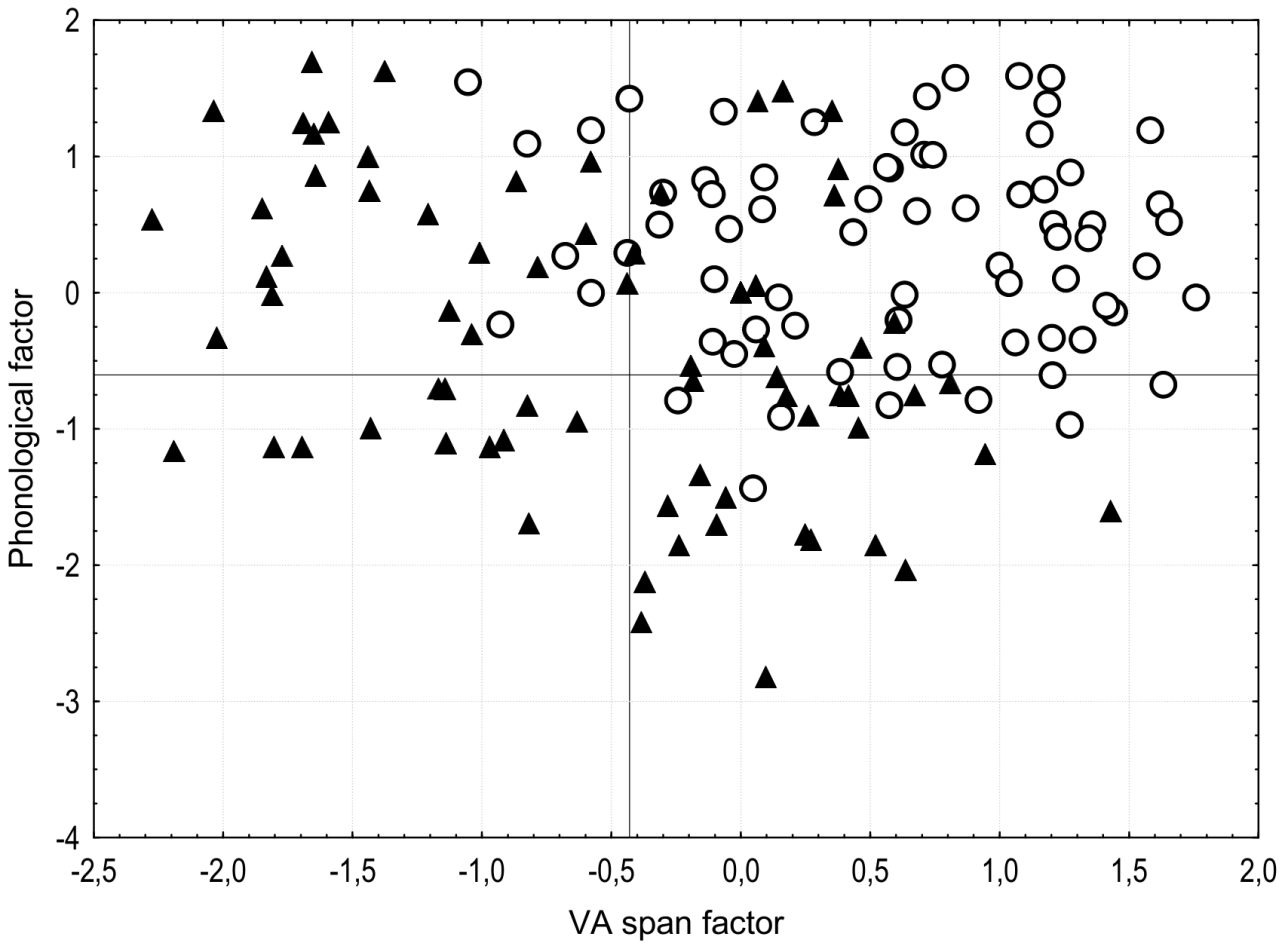
Scatterplot of the participants according to their factorial coefficients. Scatterplot of the dyslexic (triangle) and control participants (empty circle) according to their visual attention (VA) span and phonological factorial coefficients.

In the above analyses, two distinct cognitive processes have been identified that independently contribute to the reading performance of dyslexic and control children. The next step is to explore whether different cognitive subgroups can be identified within this homogeneous population with regard to the reading profile. For this purpose, we analyzed the distribution of the individual VA span and phonological factor coefficients derived from the principal components analysis. The dyslexic children whose score on one or the other factor fell below the 10^th^ percentile of the control group factorial coefficients were considered as having a cognitive impairment, either a VA span (−0.43 for the VA span factor) or a phoneme awareness disorder (−0.60 for the phonological factor). Figure 1 shows the scatterplot of the participants, dyslexic and control children, based on their VA span and phonological factorial coefficients. Despite their homogeneous reading profile, our group of dyslexic children splits into four cognitively-distinct subgroups. Indeed, 23 dyslexic children (32%) show a single phonological disorder, 24 (34%) show a single VA span deficit and 12 (17%) a double disorder characterized by poor phonological and VA span abilities. A remaining 17% shows none of these two cognitive deficits.

## Part 2: Focusing on subgroups with a single cognitive disorder

In Part 1, we provided evidence that a group of dyslexic children selected to have a homogeneous mixed reading profile nevertheless show a variety of cognitive disorders. This finding suggests a rather weak relationship between reading profiles and cognitive disorders. In the second part of the investigation, we will address this issue by directly comparing reading and spelling performance in the two groups of dyslexic children previously identified as having either a pure phonological disorder or a pure VA span disorder. The two groups were first matched for chronological age, reading age and single letter identification skills. We further ensured that their phonological and VA span disorder remained when compared to a younger group of control children of the same reading level. Our purpose was to compare two groups with contrasted cognitive disorders that were otherwise matched. We then focused on these two contrasted groups and carried out a quantitative and a qualitative analysis of their performance. The analysis of performance of cognitively-distinct subgroups of dyslexic children was expected to provide additional insights on the degree of transparency/opacity of the relationship between reading profiles and cognitive disorders. In particular, we were interested in potential imbalances with poorer pseudo-word than irregular word reading abilities in the group with a single phonological disorder and a reverse pattern in the group with a single VA span disorder.

### 1. Characteristics of the participants in the two contrasted subgroups

Nine children of the phonological group and 10 VA span impaired children who did not match for chronological age, reading age or single letter identification were excluded from the analysis. Fourteen children from the VA span group and fourteen matched children from the phonological group were retained together with, fourteen CA matched control children. An additional reading-age-matched control group of fourteen children was further included to establish whether the phonological and VA span deficits highlighted through comparison with children of the same chronological age further remained when compared to younger children of the same reading level. The four groups' characteristics are presented in Table 3.

**Table 3:** Scores and comparisons of the two dyslexic groups, and the two control groups.

	Dyslexics VA (a)	Dyslexics P (b)	Controls CA (c)	Controls RA (d)
Age and Tasks	Mean (SD)	Mean (SD)	Mean (SD)	Mean (SD)
	Range	Range	Range	Range
CA	120,4 (14,2)*d*	122,4 (10,8)*d*	118,6 (13,1)*d*	84,3 (3,9)*abc*
	98–146	102–142	101–141	77–88
RA	86,5 (4,2)*c*	88,1 (6,9)*c*	111,2 (15,5)*abd*	87,6 (5,6)*c*
	79–94	78–99	88–134	79–101
Phonological score	76,4 (9,4)*bd*	43,5 (12,8)*acd*	70,7 (12,9)*bd*	59,4 (18,9)*abc*
	64,4–92,8	17,8–59,4	51,1–94,4	17,8–91,7
Letter Identification	39,4 (5,3)*d*	41,6 (6,5)*d*	41,5 (6,3)*d*	30,1 (9)*abc*
	30–49	21–47	28–49	18–44
VA span score	63,4 (7,3)*bcd*	80,6 (4,3)*ad*	80,8 (7,3)*ad*	73,0 (9,3)a*bc*
	52,5–73	74,5–88,5	70,5–93,5	59,5–95

Mean and standard deviation (SD), range and comparisons (significant differences at p<.05 indicated by the group letter in italics) of the two dyslexic group, with phonological deficit (P) and with VA span deficit (VA)s, the CA matched control group and the RA matched control group on the phonological composite score, the VA composite score, and control (age and letter identification) tasks.

An ANOVA was carried out with Group as a between-subject factor (Phonological impaired group, VA span impaired group, CA controls and RA controls). A composite phonological score was computed from performance on the three phonological tasks, and a composite VA span score from the two global and partial report tasks. As shown in Table 3, the phonological disorder that characterized the phonological group in the comparison with CA controls [F(1,52) = 26.79; *p<0.001*] remained when compared to RA matched controls [F(1,52) = 9.11; *p = 0.004*]. By construction, the VA span group performed as CA controls on phoneme awareness tasks [F(1,52) = 1.18; *p = 0.282*] and slightly better than RA matched children [F(1,52) = 10.53; *p = 0.002*]. The VA span dyslexic group performed significantly lower than both CA controls [F(1,52) = 39.77; *p<0.001*] and RA controls in the VA span tasks [F(1,52) = 12.09; *p = 0.001*]. By construction, the phonological dyslexic group performed as CA controls [F(1,52) = 0.004; *p = 0.949*] and better than RA controls on the VA span tasks [F(1,52) = 7.64; *p = 0.008*]. The phonological dyslexic group and the VA span group were as efficient as CA controls in single letter processing [respectively F(1,52) = 0.003; *p = 0.957* and F(1,52) = −0.63; *p = 0.432*] and showed better performance than RA matched children [respectively F(1,52) = 19.53; *p<0.001* and F(1,52) = 12.77; *p<0.001*]. Note that lower ability of the younger RA matched control group to identify single letters might account for their relatively low VA span performance, whereas poor VA span performance in the VA span dyslexic group was found despite very good single letter processing skills. Overall these findings attest the existence of a selective phonological disorder in one dyslexic group and a selective VA span disorder in the other group. These cognitive disorders remain in the comparison with younger children of the same reading age, thus leading to exclude any interpretation in terms of reading delay.

### 2. Quantitative analysis

#### 2.1. Material and predictions

Performance of the two phonologically impaired and VA span impaired dyslexic groups and CA controls was compared for the regular word, irregular word and pseudo-word reading tasks previously described in Part 1. Two additional tasks of single word and pseudo-word spelling were administered. Children were asked to spell a list of 66 words [53]. The list included 22 regular words that could be spelled correctly by application of the most frequent phoneme-grapheme conversion rules, 22 irregular words including an inconsistent phoneme associated to a relatively infrequent grapheme and 22 exception words including an orthographic particularity or a rare grapheme which could not be inferred from the phonemic analysis of the oral input. The three types of words were matched in length and frequency. Regular, irregular and exception word frequency was 126, 131 and 134 per million respectively (from MANULEX, a database from French elementary school-readers [55]. The 66 words were randomly mixed, then dictated in a fixed order. Pseudo-word spelling was assessed using two lists of 10 bi-syllable and 10 tri-syllable pseudo-words from the ODEDYS battery [46]. The items were legal pseudo-words with no lexical neighbors.

We reasoned that if reading profiles were relevant to distinguish cognitively distinct subgroups, the two groups of children with contrasted cognitive disorders should differ in reading. Indeed, although the dyslexic participants were a priori selected to show both poor irregular word and poor pseudo-word reading performance, opposite word/pseudo-word imbalances were expected in the two contrasted subgroups of children. Lower pseudo-word reading performance was expected in the phonologically impaired group than in the VA span impaired group whereas VA span impaired dyslexic children might show lower scores in irregular word reading as compared to their phonologically impaired peers. We further extended the comparison to spelling performance. Indeed, if theoretical models of reading predict that performance in both pseudo-word and irregular word reading should be affected by either a phonological disorder or a VA span disorder, predictions slightly differ in spelling. Indeed, whereas both disorders should affect irregular word spelling, pseudo-word spelling might be differently affected as far as it mainly relies on phonological skills (i.e., the ability to identify the pseudo-word constitutive phonemes and knowledge of phoneme-grapheme mappings).

#### 2.2. Results of the quantitative analysis

**Figure 2:**
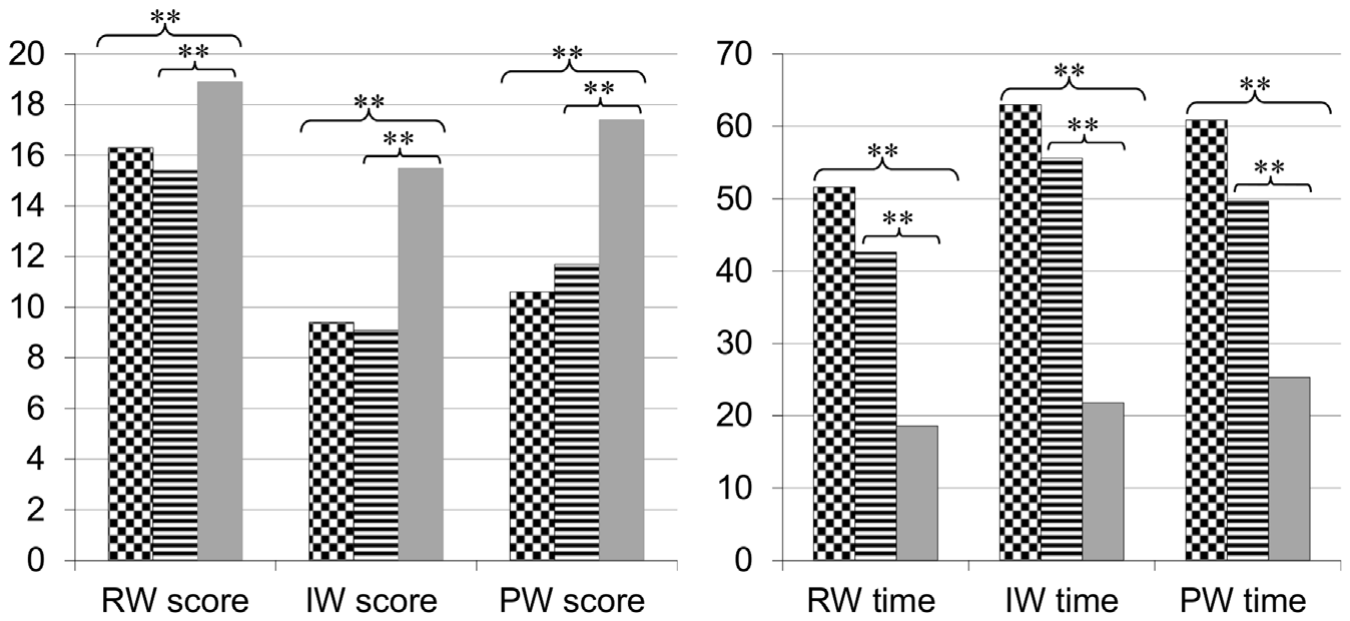
Performances of the dyslexic and control groups on reading tasks. Number of correct responses (max = 20) and reading times (in seconds) on reading tasks for dyslexics with a VA span deficit (checker board) or a phonological deficit (lines), and chronological age (CA) controls (grey) on regular words (RW), irregular words (IW) and pseudo-words (PW). **p<0,001 between CA controls and both dyslexics groups.

**Figure 3:**
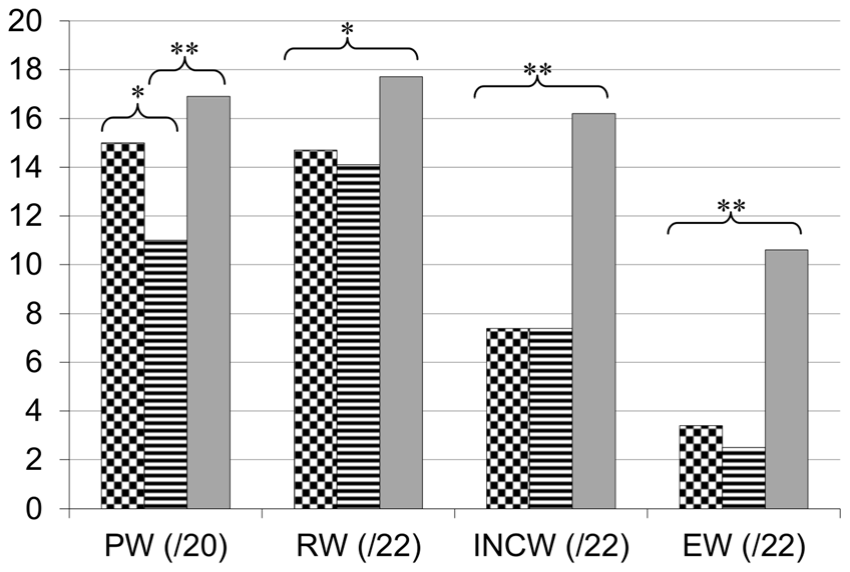
Performance of the dyslexic and control groups on the spelling tasks. Correct responses for dyslexics with visual attention span deficit (checker board), dyslexics with phonological deficit (lines), and chronological age (CA) controls (grey) on pseudo-word (PW), regular word (RW), inconsistent words (INCW) and exception words (EW) stimuli for spelling tasks. *p<0,01; **p<0,001 between CA controls and both dyslexics groups.

An ANOVA with group as the between-subject factor was carried out to compare the two dyslexic groups and each dyslexic group with the CA control group. Results are presented in Figure 2 and Figure 3 (see also Table S2 for more details). With respect to reading, the two dyslexic groups differ from CA controls by lower reading accuracy performance and longer reading times. However, the two dyslexic groups show very similar performance in word reading (accuracy and speed). In particular, they do not differ in irregular word reading and show very impaired performance on these items whatever the associated phonological or VA span disorder. Surprisingly, VA span impaired dyslexic children show the same low pseudo-word (accuracy and speed) reading performance as phonologically impaired children. For spelling, as previously for reading, the two groups do not differ in word spelling performance. In particular, the VA span group does not show lower performance on the irregular or exception words. However, the two dyslexic groups do differ in pseudo-word spelling with lower performance for the group with an associated phonological disorder. Interestingly, comparison with the control group revealed that pseudo-word spelling was preserved in the dyslexic group with a VA span disorder but impaired in the phonological group.

### 3. Qualitative analysis

#### 3.1. Material and Predictions

We analyzed reading and spelling errors to explore whether contrasted cognitive disorders resulted in distinct error patterns. The errors were divided into seven mutually exclusive categories in reading, six in spelling. The different categories of errors are presented in Table 4.

**Table 4:** Example of the error types considered in reading and spelling.

Error types	Examples
*In Reading*	
Voice-voiceless substitutions (f/v, t/d, p/b, k/g, ch/j, s/z)	JALOUX read /chalou/ not /jalou/
Regularizations	FEMME read /fèm/ not /fam/
Omissions	ESCROC read /esco/ not /escro/
Letter order errors	SOIF read /fois/ not /soif/
Parsing errors	AVANIE read /avan-ni/ not /avani/
Contextual errors	CARGO read /carjo/ not/cargo/
Paralexias	CARGO “cargo” read /escargo/ “snail”
*In Spelling*	
Voice-voiceless substitutions (f/v, t/d, p/b, k/g, ch/j, s/z)	/carp/ written GARPE not CARPE
Phonologically plausible errors	/fam/ written FAME not FEMME
Final schwa additions	/mirwar/ written MIROIRE not MIROIR
Final schwa omissions	/verb/ written VERB not VERBE
Contextual errors	/jenti/ written GANTI (/genti/) not GENTIL
Illegal sequences	/mirwar/ written MIROIRR not MIROIR
Letter order errors	/frit/ written FIRTE not FRITE

Phonological errors (i.e., voice-voiceless substitutions) in both reading and spelling were expected in the context of a phonological disorder while regularization errors in reading and phonologically plausible errors in spelling should predominate when dyslexia relates to a single VA span deficit but preserved phonological skills. Based on previous findings from Martial's case study [42], we further expected a dysfunction in the allocation of attention to the letter string of the whole word or pseudo-word orthographic units to yield specific errors in reading. A difficulty to simultaneously process all the letters of multi-letter graphemes should yield grapheme parsing errors due to splitting these units' letter-string into shorter graphemes. A VA span reduction should also limit processing of the graphemes' surrounding context thus leading to errors on contextual sensitive graphemes (s/z errors were excluded because such errors could follow from voiced-voiceless confusions as well). Partial decoding of the letter string could further result in letter omissions and a difficulty to code letter order within strings. However while graphemic parsing errors should be relatively specific, the other error types (i.e., omissions, letters order and contextual errors) are more ambiguous and could follow from a phonological disorder as well. We further hypothesized that partial decoding and poor letter order processing might result in a tendency for reporting the target word as another visually similar and more frequent real word as previously reported in some cases of visual attention dyslexia [56].

Specific errors were also expected in spelling. If a VA span reduction prevents normal development of orthographic knowledge (see [45] for supporting evidence), then the disorder should prevent normal extraction of orthographic statistical regularities then leading to higher probability for the production of illegal sequences as first described in [54] and poor knowledge of purely orthographic constraints which control for the final schwa adjunction in French. Contextual errors were further expected in spelling as in reading, assuming that normal acquisition of contextually sensitive relationships between orthographic and phonological units requires a VA span large enough to process the contextual grapheme and its surrounding context simultaneously. However as for reading, contextual errors may also reflect a phonological disorder and poor knowledge of more complex grapheme-phoneme relationships. Letter order errors in spelling were expected to primarily result from poor phonological skills.

The rate of phonologically plausible errors (PPE) in spelling was further documented using either a strict or a lax criterion. According to the strict criterion, an error was classified as phonologically plausible whenever the written word sounded as the dictated word when applying strict grapheme-phoneme conversion rules. Following this criterion, the phoneme /s/ had to be converted into “SS” between two vowels and “TE” was expected to translate the phoneme /t/ at the end of French words. According to the lax criterion, graphemes that could be pronounced as the dictated phonemes without consideration for mapping frequencies or orthographic context were considered as phonologically plausible (/s/ written “S” was then considered as phonologically plausible even in the two vowel context, and “T” was considered as plausible to translate the final phoneme /t/). We reasoned that these two categories of phonologically plausible errors both reflect good phonological processing skills. Indeed, even expanded phonologically plausible errors (lax criterion) require good ability to segment spoken words into phonemes but without consideration for the conventional orthographic rules.

#### 3.2. Results of the qualitative analysis

**Figure 4:**
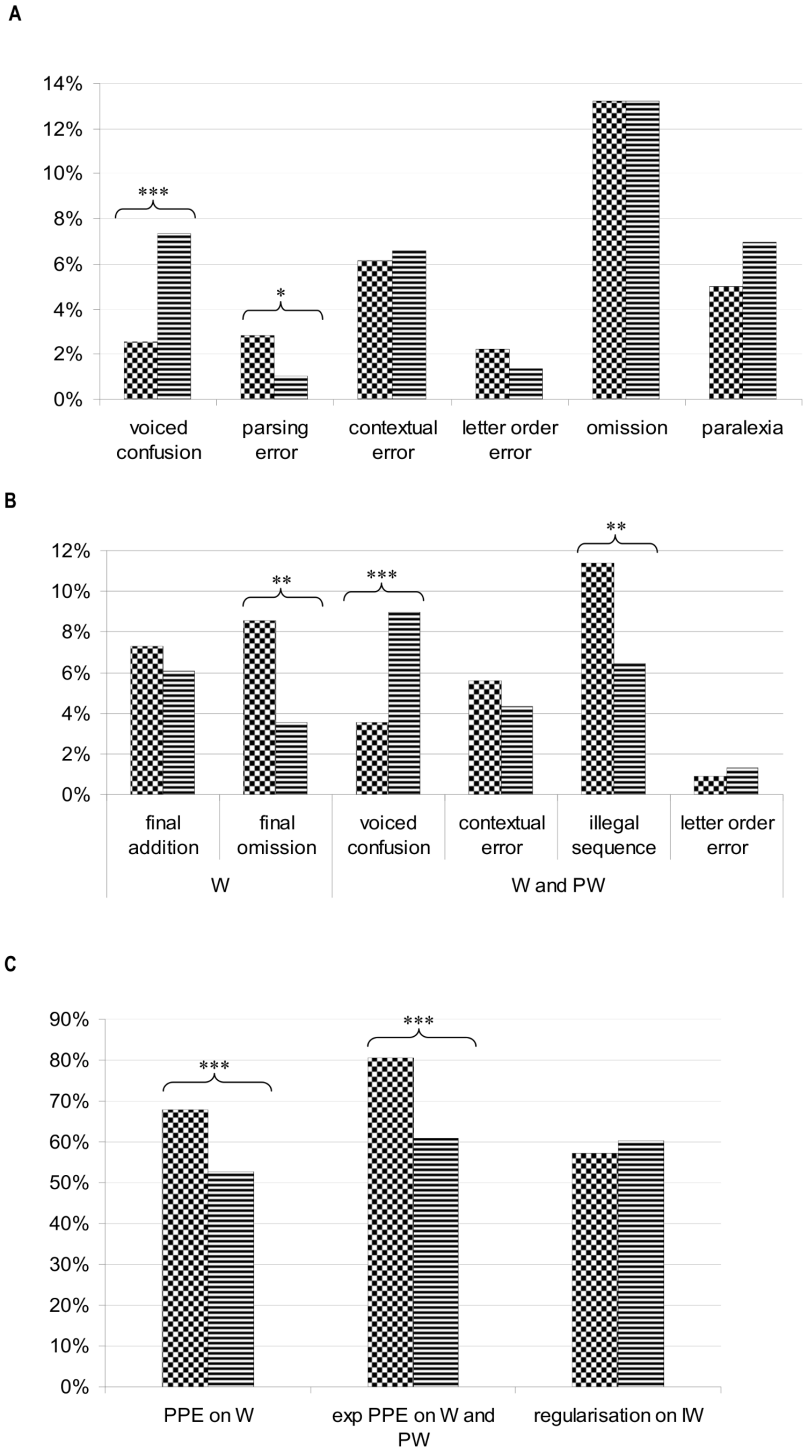
Percentage of each type of errors for both dyslexic groups. Percentage of each type of errors for dyslexics with visual attention span deficit (checker board) and dyslexics with phonological deficit (lines), on reading tasks (A), on spelling tasks (B) and phonologically plausible errors on spelling tasks (PPE and expanded PPE) and on irregular word reading task (C). *p<0,05; **p<0,01; ***p<0,001.

The error analysis was restricted to those words and pseudo-words that included a single error type, all other errors were considered as complex. The proportion of errors in each category is shown for each group in Table 5 and Figure 4. Chi-square tests were used to compare reading and spelling error rates between the phonological and the VA span dyslexic group. In reading tasks, results show that confusions between voiced/voiceless consonants are more frequent in phonologically impaired dyslexic children and quite rare in the VA span impaired group. Conversely, the dyslexic children with a VA span deficit produced more grapheme parsing errors, than the dyslexic children with a phonological deficit. Omissions, letter order errors, and contextual errors occurred in similar proportions in the two dyslexic groups. Likewise, the proportion of formal paralexias and regularization errors on irregular words was similar in the VA span impaired and phonologically impaired groups. In spelling as in reading, voiced/voiceless confusion errors were more frequent in phonologically impaired dyslexic children than in the group with a VA span deficit. However, more illegal sequences and more omissions of the final schwa were found on words for the VA span group as compared to the phonological group. Results further showed that PPE and expanded PPE were more frequent in the group of children with a VA span deficit than in children with a phonological deficit. The two groups did not differ in the rate of final additions, contextual errors, or letter order errors.

**Table 5:** Comparison of the two dyslexic groups on error types.

Error type	Dys VAS		Dys P		Chi2 (1)
*Reading*					
Voice-voiceless confusions	17/665	2,6%	49/665	7,4%	16,33***
Parsing errors	19/665	2,9%	7/665	1,1%	5,65*
Contextual errors	41/665	6,2%	44/665	6,6%	0,11
Letter order errors	15/665	2,3%	9/665	1,4%	1,53
Omissions	88/665	13,2%	88/665	13,2%	0,00
Visual paralexias on words	21/403	5,2%	28/432	6,5%	0,61
Regularizations on IW	171/298	57,4%	183/304	60,2%	0,49
*Spelling*					
Final schwa additions on words	34/466	7,3%	29/477	6,1%	0,56
Final schwa omissions on words	40/466	8,6%	17/477	3,6%	10,46**
Voice-voiceless confusions	19/536	3,5%	54/603	9,0%	13,85***
Contextual errors	30/536	5,6%	26/603	4,3%	1,00
Illegal sequences	61/536	11,4%	39/603	6,5%	8,55**
Letter order errors	5/536	0,9%	8/603	1,3%	0,39
PPE on words	316/466	67,8%	252/477	52,8%	22,09***
Expanded PPE on words	418/466	89,7%	361/477	75,7%	32,24***
Expended PPE on PWs	14/70	20,0%	7/126	5,6%	9,81*

Comparisons of the two dyslexic groups, with phonological deficit (P) and with visual attention span deficit (VA), on the rate of each error types in reading and spelling tasks.

*** p<.001 ** p<.01 *p<.05.

## Discussion

The well-recognized heterogeneity of the dyslexic population led researchers to propose classifications based on reading profiles [8], [57], [22], [58], [24]. Different reading profiles were thought to distinguish subsets of dyslexic children with different cognitive disorders. However, the classification based on reading profiles yielded a relatively poor description of the dyslexic population [59]. Indeed, a deficiency in phoneme awareness and phonological processing tasks was reported not only in phonological but also in surface profiles [28], [24], [60], [26]. Moreover, reading-based subtypes did not show stability over time thus suggesting they did not reliably characterize cognitively distinct subgroups [61]. Accordingly, the existence of developmental subtypes was denied and the tendency was to treat developmental dyslexia as a unitary syndrome with a phonological deficit as the proximal cause [33], [62], [24], [32], [63].

Evidence for a failure of reading profile subtypes to account for the heterogeneity of developmental dyslexia was interpreted as evidence against dyslexia subtypes. However, such a failure might reflect the irrelevance of the reading-profile-based subtypes rather than being strong evidence against cognitively-based subtypes. Recent evidence that some children with developmental dyslexia exhibit a visual attention span disorder that typically dissociates from phonological problems [2] and taps attentional brain regions while leaving preserved those associated with phonological processing [53], [41], [5] put the subtype issue back on the front row.

In this paper, we focused on mixed developmental dyslexia, a condition characterized by poor pseudo-word and poor regular and irregular word reading. Despite its severity and its high incidence in the dyslexic population (two third of cases), very few studies investigated this form of developmental dyslexia (see however [42]). The 71 dyslexic children who participated showed very poor performance on all types of items (regular or irregular words and pseudo-words) either considering reading accuracy or reading speed. This is potentially an important issue as reading latency may be more prone than accuracy measures to reflect a reading disorder, at least in more transparent languages than English [24], [27]. This group of children was very severely impaired since demonstrating a 38 months delay in reading for an average chronological age of 10 years 5 months. They were administered tasks of phoneme awareness and global/partial letter report to respectively assess their phonological and visual attention span skills, two independent cognitive skills involved in reading acquisition [2], [43], [37]. Our purpose was to focus on the cognitive underpinnings of developmental dyslexia as defined within the framework of the multi-trace memory model [36] and for this reason, only the phonological and VA span mechanisms which are basic mechanisms of the reading system were taken into account.

We first assessed whether children of this group - which was homogeneous with respect to reading profile - showed the same cognitive disorder or whether the group was cognitively heterogeneous. We then explored whether distinct and unrelated cognitive disorders translated in different reading and spelling patterns.

### 1. One reading profile, multiple cognitive disorders

Although our dyslexic participants exhibited poor phoneme awareness skills and poor VA span abilities as a group, only a few participants (12%) showed a double deficit. In fact, a majority of children exhibited either a single phonological disorder (32%), or a single VA span disorder (34%), two independent cognitive factors which were further found to independently contribute to all reading measures. These findings are clear evidence that children with a similar mixed reading profile do not form a homogeneous population with respect to the associated cognitive disorder.

Surprisingly, the current findings largely replicate those reported by [2] whereas the later study was conducted on an unselected population of French dyslexic children who did exhibit a variety of reading profiles. Not only is our population of mixed dyslexic children heterogeneous at the cognitive level but further, incidence of a double deficit in this population is not higher than in previous studies conducted on unselected groups of either French or English dyslexic participants [2]. This finding undermines the classical explanation of mixed dyslexia [22] according to which this condition would follow from two independent disorders, one involved in the build-up of the phonological procedure (responsible for phonological dyslexia), the other one preventing normal orthographic knowledge acquisition (as seen in surface dyslexia). Such a view would have predicted a majority of double deficits in the mixed dyslexia population, cumulating the phonological disorder typically described in children with poor pseudo-word reading [10], [12], [14] and the VA span disorder reported in cases with poor orthographic knowledge [39], [15].

Our results show to the contrary that even severely impaired children with mixed dyslexia suffer, for more than 60% of them, from an isolated cognitive disorder: a phonological deficit or a VA span deficit. The self-teaching theory provides a straightforward account of the impact of a single phonological disorder on the building-up of the reading system [29]–[31], [64]. This theory assumes that the ability to translate unfamiliar printed words into their spoken equivalents is the main way by which orthographic knowledge is acquired [65]–[68]. The self-teaching theory thus predicts that poor phonological skills preventing normal acquisition of letter-sound mapping would disturb phonological recoding of unfamiliar words, thus leading to poor pseudo-word reading. In turn, poor phonological decoding would compromise normal development of word-specific orthographic knowledge, alter irregular word reading and thus lead to the mixed dyslexia profile.

However, a main finding of the current study is to show that a mixed reading profile can be associated with a single VA span disorder (see [42], for similar findings in a single case study). The MTM model [36] provides a straightforward account of this finding. Indeed within this framework, a VA span disorder that hampers the entire letter sequence of most words to be processed in a single step would primarily affect irregular word reading [37]. However, analytic processing requires the VA span to be large enough to process in parallel all the letters of relevant orthographic units (such as multi-letter graphemes or syllables). It follows that a VA span impairment can result not only in difficulties for processing words globally but it will further prevent normal processing of multi-letter orthographic units, thus leading to the word and pseudo-word reading difficulties that characterize mixed dyslexia. The MTM model thus predicts a causal relationship between VA span deficit and dyslexia, as evidenced through simulations showing that a VA span reduction impacts reading performance independently of the model's phonological processing [36]. Further support for a causal relationship comes from evidence that intensive VA span-based intervention results in higher VA span abilities, increased reading performance and specific brain activity modulations [69].

Another main finding of the current study is to provide a straightforward account of the high incidence of mixed reading profiles in the dyslexic population. We indeed show here that mixed reading profiles can be associated with either a single phonological disorder, or a single VA span disorder or a double deficit, and potentially some other, not yet identified, cognitive disorder able to account for the group of children with none of the phonological or VA span disorder (it is worth noting that the double deficit group does not differ from the single deficit groups in reading delay). This suggests that the mixed reading profile may have different causes, which would in turn yield higher prevalence in the dyslexic population. More generally, the current findings confirm the prevalence of isolated cognitive deficits in the dyslexic population and the cognitive heterogeneity of developmental dyslexia [2].

### 2. Different cognitive disorders, subtle reading and spelling differences

In the second part of this paper, we focused on the two groups of dyslexic children previously identified as having a single phonological or VA span disorder. The two groups were matched on chronological age and reading age, to ensure that any observed difference could not be attributed to differences in print exposure or neural maturation. We further compared the phonological and VA span performance of the two groups with that of a new reading age-matched control group to demonstrate that their underlying cognitive disorder was not just the consequence of their poor reading skills [70]. The phonological dyslexic group showed a selective phonological processing disorder relative to the two control groups. This finding is well in agreement with previous evidence that phonological awareness scores in developmental dyslexia typically lag behind those of younger but typical children matched for reading age [25], [22]. This also emerged from the comparison of the VA span dyslexic group and the reading age control group. Indeed, results clearly showed that VA span impaired dyslexic children had a more reduced VA span than younger controls of the same reading level. Lower performance than reading age-matched controls excludes any effect of the level of reading attained by our dyslexic participants and may rather suggest a causal relationship between VA span abilities and reading performance. Previous studies have already suggested that VA span abilities are not just the consequence of the children's poor reading performance [15], [71].

We reasoned that comparison of the reading and spelling performance patterns of the two dyslexic groups would provide insights on the consequences of each of the phonological and VA span disorder on the establishment of the reading system. In particular, based on the fact that the difficulties experienced by dyslexic individuals in reading pseudo-words are generally explained in terms of their poor phonological skills (especially in phoneme awareness), we expected children from the phonological group to show lower pseudo-word reading and spelling performance relative to the VA span impaired dyslexic group.

Against the view that different cognitive disorders would yield imbalances in irregular word and pseudo-word reading, our two groups of dyslexic children despite contrasted cognitive disorders were found to only very slightly differ in reading and spelling. It is first noteworthy that their accuracy performance on words (in particular for the irregular, inconsistent and exception words) was very similarly low in both reading and spelling. The two groups did not differ either in regular and irregular word reading latency. This result suggests that poor phonological skills or poor VA span abilities similarly impact orthographic knowledge acquisition. Although the role of phonology in orthographic learning is well documented [72], [73], [31], the potential role of VA span has received little attention. As indirect evidence, Bosse et al. [45] showed that the orthographic form of new words is better memorized in a self-teaching paradigm when the entire word orthographic information is available for visual processing at once than when the word sublexical units are discovered in turn one at a time. In line with this result, the current findings suggest that beyond phonological skills and decoding abilities, VA span abilities may play an important role in word orthographic learning.

Another unexpected finding concerns pseudo-word processing. The two groups show very low pseudo-word reading accuracy and speed performance and no relative imbalance in favor of the VA span group. First, these findings reveal an asymmetric relation between phonological disorders and pseudo-word reading. While a phonological disorder results in poor pseudo-word reading accuracy and slow pseudo-word reading speed, poor pseudo-word performance (accuracy and speed) does not necessarily reflect a phonological disorder. This is a very important issue as there is a very strong tradition in the literature that considers a disorder in pseudo-word reading as the hallmark of a phonological deficit [74]–[76], [62]. Second, our results suggest that a VA span disorder can affect pseudo-word reading as a phonological disorder does. In line with this finding, VA span abilities in typical readers have been shown to contribute to pseudo-word reading performance independently of children's phonological skills [43]. Furthermore, some studies have reported cases of surface or mixed reading profiles which were associated with slow pseudo-word reading but preserved phonological skills [42], [17], [15], [77], suggesting that a non-phonological disorder might affect pseudo-word reading speed. It is clear from the current study (see also [2]–[43]) that a single VA span disorder contributes to poor pseudo-word reading (accuracy and speed). This is quite compatible with the MTM prediction [36] that VA span allows grapheme identification within the pseudo-word letter string. Reduced VA span abilities yield dyslexic children to process shorter sub lexical units than their non-dyslexic peers, with direct incidence on pseudo-word reading speed. The overall data thus suggests that phonological and VA span problems both compromise normal acquisition and use of alphabetic knowledge.

Another important finding is the strong pseudo-word spelling impairment in the phonological group whereas the VA span group performs as CA matched non dyslexic children. In the absence of strong behavioral differences in reading between our two cognitively contrasted dyslexic groups, we focused on the participants' spelling performance which is typically viewed as another marker of dyslexia. As pseudo-word spelling relies on the ability to accurately parse the pseudo-word phonological string into phonemes, a phonological disorder is expected to have strong impact on pseudo-word spelling, which was found. In contrast, a VA span disorder was expected to prevent normal processing of the visual input in reading but should have no detrimental effect in spelling which primarily relies on the phonological analysis of the spoken input. In line with these expectations, pseudo-word spelling was less accurate when developmental dyslexia was associated with a phonological disorder, but the children with a VA span disorder showed normal pseudo-word spelling abilities, as expected given their preserved phonological skills.

### 3. Different error types depending on the underlying cognitive disorder

Interestingly, the qualitative analysis of reading and spelling performance provided some insights on the two groups' cognitive underpinnings. A first important issue is that confusions between voiced/voiceless consonants in either reading or spelling were more frequently observed in the phonologically impaired group but quite rare in the VA span group. Such errors may suggest a difficulty to discriminate between phonologically similar phonemes or identify phonemes appropriately, in line with the well-documented categorical perception deficit in developmental dyslexia [78], [79].

Evidence for a higher incidence of phonologically plausible errors (PPE) or expanded PPE in spelling in the VA span group provides further evidence for reliance on phonological recoding skills, well in line with the VA span group's good phonological abilities. In contrast the VA span group was more prone to produce grapheme parsing errors in reading, as expected if children cannot simultaneously process the whole grapheme letter strings due to their poor VA span abilities. It has been hypothesized that an inability to process the whole word letter string during reading would result in poor orthographic knowledge acquisition [56]. Accordingly, we found that VA span impaired children produced more illegal sequences in spelling and a higher rate of final schwa omission errors due to a disrupted analysis of orthographic statistical regularities and poor knowledge of orthographic constraints.

As a matter of fact, the main difference between the two dyslexic groups highlighted from the quantitative analysis is first that phonological skills and pseudo-word spelling are associated and second, that pseudo-word spelling performance is not impaired in the VA span group of dyslexic children. There is indeed strong evidence that pseudo-word spelling primarily relies on the ability to identify and isolate each of the spoken pseudo-word constitutive phonemes in order to activate their corresponding graphemes and provide a phonologically plausible translation of the dictated pseudo-word. In contrast, the VA span hypothesis would predict good pseudo-word spelling skills in the group of children with a single VA span disorder, which is observed.

## Conclusion

The current study questioned the relevance of reading profiles to identify cognitively homogeneous subgroups of dyslexic children. We first showed that dyslexic children selected to have the same reading profile nevertheless split into distinct cognitive subgroups. We further showed that the two subgroups characterized by distinct and independent cognitive disorders nevertheless exhibited very similar reading and spelling performance. The overall findings question the validity of reading profile subtypes as a classification method to reduce heterogeneity in the dyslexic population and define cognitively homogenous subgroups.

More and more evidence is accumulating that VA span abilities contribute to reading performance in both skilled and dyslexic readers, independently of their phonological skills. The VA span involves parietal regions known for their role in visual attention, not phonology [53], [41]. The analysis of two contrasted cases of developmental dyslexia with either a single phonological disorder or a single VA span disorder, further established that these cognitively distinct subtypes further dissociated at the neural level [5]. Available data thus supports the existence of distinct cognitively based subgroups but suggests an opaque relationship between these cognitive subgroups and their reading profiles. Future neuroimaging and genetic studies should take this issue into account since synthesizing over cognitively heterogeneous children would entail potential pitfalls.

Lastly, the present findings have important implications from a clinical perspective. Although reading profiles provide valuable information on the development of the reading system, our results clearly indicate that they provide no reliable information on the cognitive disorders involved in the reading disorder. Even if some cues about the underlying cognitive deficit can be found in reading and spelling performance, the clinician has to conduct additional investigations more directly targeted towards the identification of the associated cognitive disorders. Assessment of the child phonological skills is absolutely necessary, all the more that our results indicate that poor pseudo-word reading is not a sufficient clue to conclude that an underlying phonological disorder is at play. Investigation of the child VA span abilities is further required as more and more evidence suggests a specific contribution of this component to normal and atypical reading and spelling [2], [43], [45], [80]. Assessment of the phonological and VA span abilities of dyslexic children is a very important issue in clinical practice. This step is indeed crucial to identify which remediation program is more appropriate to improve the child's reading performance.

## Supporting Information

Table S1 **Correlations.** Correlations among Chronological Age (CA), Letter Identification (Letter Id.), Deletion, Segmentation (Segment.), Acronyms, Whole report, Partial report, Reading Age (RA), Regular word (RW) reading score and time, Irregular word (IW) reading score and time, Pseudo-word (PW) reading score and time, and partial correlations (controlling for chronological age and letter identification) below the diagonal (N = 142). * p<.00025 (Bonferroni correction).
(DOCX)

Table S2 **Scores and comparisons of the two dyslexic groups and the two control groups.** Scores and comparisons of the two dyslexic groups and the chronological age (CA) matched control group in word reading (accuracy and speed), and spelling of regular words (RW), irregular words (IW), pseudo-words (PW), inconsistent words (INCW) and exception words (EW).
(DOCX)
