# Supplementary material for: New Insights on Developmental Dyslexia Subtypes: Heterogeneity of Mixed Reading Profiles
Source: PLoS One. 2014 Jun 11;9(6):e99337. doi: 10.1371/journal.pone.0099337 (PMC4053380; doi:10.1371/journal.pone.0099337)
Supplement: Table S1 — Correlations. Correlations among Chronological Age (CA), Letter Identification (Letter Id.), Deletion, Segmentation (Segment.), Acronyms, Whole report, Partial report, Reading Age (RA), Regular word (RW) reading score and time, Irregular word (IW) reading score and time, Pseudo-word (PW) reading score and time, and partial correlations (controlling for chronological age and letter identification) below the diagonal (N = 142). * p<.00025 (Bonferroni correction). (DOCX) [file pone.0099337.s001.docx]

|  | | Letter Id. | | Deletion | Segment. | Acronym | Wh. report | Part. report | RA | RW score | RW time | EW score | EW time | PW score | PW time |
| --- | --- | --- | --- | --- | --- | --- | --- | --- | --- | --- | --- | --- | --- | --- | --- |
| CA | | .05 | | .00 | -.02 | .09 | .26 | .22 | -.20 | .20 | -.20 | .32* | -.22 | .10 | -.17 |
| Letter Id. | | -- | | .03 | -.12 | .08 | .45* | .43* | -.29 | .19 | -.27 | .22 | -.29 | .23 | -.28 |
| Deletion | |  | | -- | .41* | .47* | .20 | .07 | -.35* | .43* | -.30* | .48* | -.31* | .38* | -.25 |
| Segment. |  | | | .41* | -- | .38* | -.05 | -.13 | -.10 | .22 | -.01 | .22 | -.03 | .11 | .05 |
| Acronyms | |  | | .47* | .39* | -- | .15 | .04 | -.37* | .44* | -.32* | .44* | -.30* | .38* | -.24 |
| Wh. report | | |  | .22 | .01 | .12 | -- | .71* | -.63* | .50* | -.63* | .61* | -.62* | .51* | -.62* |
| Part. report | | |  | .06 | -.09 | -.01 | .61* | -- | -.54* | .44* | -.54* | .54* | -.51* | .50* | -.53* |
| RA | |  | | -.36* | -.15 | -.35* | -.56* | -.46* | -- | -75* | .94* | -.86* | .93* | -.80* | .89* |
| RW score | |  | | .45* | .25 | .43* | .45* | .38* | -.73* | -- | -.74* | .82* | -.68* | .85* | -.65* |
| RW time | |  | | -.31* | -.05 | -.30 | -.57* | -.47* | .93* | -.72* | -- | -.83* | .98* | -.70* | .95* |
| IW score | |  | | .51* | .28 | .43* | .55* | .47* | -.85* | .80* | -.81* | -- | -.82* | .80* | -.74* |
| IW time | |  | | -.32* | -.07 | -.28 | -.55* | -.42* | .92* | -.65* | .97* | -.80* | -- | -.65* | .95* |
| PW score | |  | | .39* | .14 | .37* | .46* | .45* | -.79* | .84* | -.68* | .81* | -.63* | -- | -.64* |
| PW time | |  | | -.25 | .01 | -.22 | -.56* | -.45* | .88* | -.62* | .95* | -.72* | 0.94* | -.61* | -- |
