# Supplementary material for: New Insights on Developmental Dyslexia Subtypes: Heterogeneity of Mixed Reading Profiles
Source: PLoS One. 2014 Jun 11;9(6):e99337. doi: 10.1371/journal.pone.0099337 (PMC4053380; doi:10.1371/journal.pone.0099337)
Supplement: Table S2 — Scores and comparisons of the two dyslexic groups and the two control groups. Scores and comparisons of the two dyslexic groups and the chronological age (CA) matched control group in word reading (accuracy and speed), and spelling of regular words (RW), irregular words (IW), pseudo-words (PW), inconsistent words (INCW) and exception words (EW). (DOCX) [file pone.0099337.s002.docx]

|  | Dys VA (a) |  | Dys P (b) |  | CA (c) |  | a vs. b |  | a vs. c |  | b vs. c |  |
| --- | --- | --- | --- | --- | --- | --- | --- | --- | --- | --- | --- | --- |
| Age and Tasks | Mean (SD) | Range | Mean (SD) | Range | Mean (SD) | Range | F (2,39) | *p* | F (2,39) | *p* | F (2,39) | *p* |
| *Reading* | | | | | | | | | | | | |
| RW score (/20) | 16,3 (2,2) | 9 - 20 | 15,4 (2,3) | 9 - 20 | 18,9 (0,8) | 14 - 20 | 1,34 | *0,254* | 13,88 | *0,000* | 23,85 | *0,000* |
| RW time (sec) | 51,6 (20) | 21 - 105 | 42,6 (17) | 22 - 80 | 18,6 (5,4) | 10 - 38 | 2,39 | *0,130* | 31,91 | *0,000* | 16,83 | *0,000* |
| IW score (/20) | 9,4 (3,7) | 1 - 17 | 9,1 (2,4) | 3 - 18 | 15,5 (2,4) | 7 - 20 | 0,04 | *0,845* | 32,20 | *0,000* | 34,47 | *0,000* |
| IW time (sec) | 63 (30,7) | 22 - 149 | 55,6 (23,9) | 21 - 105 | 21,8 (8,3) | 11 - 46 | 0,72 | *0,400* | 22,53 | *0,000* | 15,18 | *0,000* |
| PW score (/20) | 10,6 (1,7) | 6 - 15 | 11,7 (2) | 8 - 17 | 17,4 (1,2) | 14 - 19 | 2,74 | *0,106* | 115,31 | *0,000* | 82,48 | *0,000* |
| PW time (sec) | 60,9 (21,5) | 30 - 127 | 49,7 (17,8) | 24 - 97 | 25,3 (6,3) | 14 - 39 | 3,22 | *0,080* | 32,40 | *0,000* | 15,19 | *0,000* |
| *Transcription* | | | | | | | | | | | | |
| PW (/20) | 15 (2,5) | 10 - 19 | 11 (4,2) | 3 - 16 | 17,7 (2,2) | 14 - 21 | 11,59 | *0,002* | 2,69 | *0,109* | 25,46 | *0,000* |
| RW (/22) | 14,7 (3,3) | 10 - 21 | 14,1 (2,6) | 9 - 19 | 16,2 (3,1) | 11 - 21 | 0,39 | *0,535* | 8,51 | *0,005* | 12,55 | *0,001* |
| INCW (/22) | 7,4 (3,4) | 2 - 12 | 7,4 (2,5) | 3 - 12 | 10,6 (4,7) | 4 - 21 | 0,01 | *0,950* | 60,33 | *0,000* | 59,36 | *0,000* |
| EW (/22) | 3,4 (2,8) | 0 - 9 | 2,5 (2,3) | 0 - 7 | 16,9 (2,3) | 10 - 19 | 0,44 | *0,512* | 31,01 | *0,000* | 38,82 | *0,000* |
